# Advances in Management of Bladder Cancer—The Role of Photodynamic Therapy

**DOI:** 10.3390/molecules27030731

**Published:** 2022-01-23

**Authors:** Tomasz Kubrak, Michał Karakuła, Marcin Czop, Aleksandra Kawczyk-Krupka, David Aebisher

**Affiliations:** 1 Department of Biochemistry and General Chemistry, Medical College of the University of Rzeszów, 35-310 Rzeszów, Poland; 2 Department of Analytical Chemistry, Medical University of Lublin, Chodźki 4a, 20-093 Lublin, Poland; michal.karakula@umlub.pl; 3 Department of Clinical Genetics, Medical University of Lublin, Radziwiłłowska 11, 20-080 Lublin, Poland; marcin.czop@umlub.pl; 4 School of Medicine with the Division of Dentistry in Zabrze, Department of Internal Diseases, Angiology and Physical Medicine, Center for Laser Diagnostics and Therapy, Medical University of Silesia in Katowice, Batorego Street 15, 41-902 Bytom, Poland; akawczyk@gmail.com; 5 Department of Photomedicine and Physical Chemistry, Medical College of the University of Rzeszów, 35-310 Rzeszów, Poland; daebisher@ur.edu.pl

**Keywords:** bladder cancer, PDT, photodynamic therapy, diagnosis, cancers

## Abstract

Photodynamic therapy (PDT) is a non-invasive and modern form of therapy. It is used in the treatment of non-oncological diseases and more and more often in the treatment of various types of neoplasms in various locations including bladder cancer. The PDT method consists of local or systemic application of a photosensitizer, i.e., a photosensitive compound that accumulates in pathological tissue. Light of appropriate wavelength is absorbed by the photosensitizer molecules, which in turn transfers energy to oxygen or initiates radical processes that leads to selective destruction of diseased cells. The technique enables the selective destruction of malignant cells, as the photocytotoxicity reactions induced by the photosensitizer take place strictly within the pathological tissue. PDT is known to be well tolerated in a clinical setting in patients. In cited papers herein no new safety issues were identified. The development of anti-cancer PDT therapies has greatly accelerated over the last decade. There was no evidence of increased or cumulative toxic effects with each PDT treatment. Many modifications have been made to enhance the effects. Clinically, bladder cancer remains one of the deadliest urological diseases of the urinary system. The subject of this review is the anti-cancer use of PDT, its benefits and possible modifications that may lead to more effective treatments for bladder cancer. Bladder cancer, if localized, would seem to be a good candidate for PDT therapy since this does not involve the toxicity of systemic chemotherapy and can spare normal tissues from damage if properly carried out. It is clear that PDT deserves more investment in clinical research, especially for plant-based photosensitizers. Natural PS isolated from plants and other biological sources can be considered a green approach to PDT in cancer therapy. Currently, PDT is widely used in the treatment of skin cancer, but numerous studies show the advantages of related therapeutic strategies that can help eliminate various types of cancer, including bladder cancer. PDT for bladder cancer in which photosensitizer is locally activated and generates cytotoxic reactive oxygen species and causing cell death, is a modern treatment. Moreover, PDT is an innovative technique in oncologic urology.

## 1. Introduction

Bladder cancer is one of the deadliest urological diseases urinary system. Urothelial cancer is the most common type of bladder cancer. This type is present in 90% of bladder cancer patients. Squamous cell carcinoma and adenocarcinoma, although less frequently (5% and 2%, respectively) are associated with advanced stages and have a higher mortality rate than urothelial carcinoma [1].

The World Health Organization ranks bladder cancer the ninth most commonly diagnosed cancer. Men have a higher incidence and it is the 13th most common cause of cancer death in the world. Bladder cancer is the most expensive malignant tumor due to the cost of treatment [2,3,4,5].

Generally, patients with bladder cancer are divided into two histopathological stages, namely: 70% of cases are mucosal cancer without damage to the basement membrane (non-invasive bladder cancer or NMIBC; stage Ta—lowest stage), the remaining 30% are associated with invasive cancer that affects basement membrane (T1) or muscle (muscle infiltrating bladder cancer or MIBC; stage T2–T4). In the latter group of patients, unpredictable metastases may occur. The MIBC stage remains difficult to heal. About half of the patients, according to the five-year rate survive and recover completely [6,7].

About 70% of newly diagnosed bladder cancer cases are superficial and belong to the NMIBC stage. Once healed, these cases usually have a high risk of recurrence. Of patients who experience relapse, 20–40% may progress to the MIBC stage, after which they become subject to ineffective therapies. These circumstances indicate that an effective NMIBC therapy strategy is absolutely required [8]. Bladder cancer is the most common condition that causes rare, non-specific symptoms that may sometimes develop asymptomatically over an extended period of time. In the treatment of bladder cancer, resection of the neoplastic fragments is most often performed. It is now known that over 70% of patients relapse after initial therapy. Treatment options are also chemotherapy and intravesical immunotherapy, mainly in patients at high risk of recurrence. Intravesical chemotherapy shows reduction of short-term tumor, but disease progression does not always change. Bladder cancer accounts for more than 3% and is one of the 10 most common types of cancer diagnosed in the world and 2.1% of all cancer deaths are due to bladder cancer. Bladder cancer is a disease with a varied natural course. Tumors are classified as low-grade, have a low rate of progression, and require initial endoscopic treatment and surveillance, but rarely pose a risk to the patient [9].

Painless hematuria is the most characteristic symptom. The course of the disease naturally depends on the location and nature of the cancer. There are both minimally invasive forms, types with low malignancy and low risk of progression, and types with high malignancy and high risk of progression and relapse. In recent years, scientists have made a significant contribution to the diagnosis and treatment of bladder cancer. Modern diagnostic methods used to detect bladder cancer include imaging (ultrasound, intravenous urography (IVU), computed tomography (CT) and magnetic resonance imaging (MRI)), cystoscopy, biopsy, and cytology. For example, cystoscopy is that considered the gold standard in the initial treatment of bladder cancer. In addition to the methods presented above, it is possible to detect bladder cancer with the use of fluorescence cystoscopy (PDD). The diagnostics of bladder cancer under the control of fluorescence is characterized by excellent sensitivity and specificity. Studies comparing white light cystoscopy and fluorescence cystoscopy confirm an increased incidence of in situ bladder cancer detection with the latter method by approximately 30%. Narrowband imaging improves the detection rate and reduces the risk of relapse after 3 and 12 months. Blue light cystoscopy (BLC) detects up to 14% of Ta/T1 papillary lesions and 40% of in situ cancer lesions omitted by conventional cystoscopy. The method of photodynamic diagnosis of neoplasms is the most accurate method of imaging neoplastic tissue and allows, in contrast with ordinary cystoscopy, to visualize all neoplastic foci in the earliest stages of their formation.

Modern fluorescence diagnostics of bladder tumors shows great potential both in basic biomedical research and in clinical practice [10,11].

In addition, ultrasound of the kidneys and bladder is useful in the initial diagnosis of some suspected bladder cancer cases. In addition, a number of urinary biomarkers are available to assist in the diagnosis and treatment of patients with non-invasive bladder cancer. Many of them have better sensitivity compared to urinary cytology but are less specific. This has proved valuable in some situations, but so far, they are not part of the standard guidelines. Biomarker tests detected in urine using fluorescence are a great hope in the diagnosis of bladder cancers. Biomarkers are compounds that are either toxic themselves or are metabolites in the urine of the toxic parent compound. In the case of bladder cancer, attention is focused on biomarkers whose exposure is marked by exposure to bladder cancer genes. They are a useful source of information as their presence indicates direct exposure of urothelium to related carcinogens as urine passes through the bladder. In addition, the material for testing (in this case urine) is easy to collect from the patient and the method of their determination is commonly practiced, which is why it is not a big problem [12]. The US Food and Drug Administration (FDA) has approved six urine biomarkers that are helpful in the diagnosis and analysis of bladder cancer. For example, nuclear matrix proteins (NMPs) are used both in the context of diagnosis and monitoring for cancer recurrence. Bladder tumor antigen assays are used for monitoring BC recurrence in combination with cystoscopy [13,14]. Transurethral resection of a bladder tumor is the first line treatment for patients. Unfortunately, this is not a sufficient technique. Therefore, other more or less advanced techniques are used. These include the use of chemotherapeutic agents, usually mitomycin-C (MMC) or immunotherapeutic agents. Intravesical agents are the standard treatment for high-grade bladder cancer and should be used as a maintenance regimen [15].

Many effective methods have been developed, including PDT, and new drugs have been used that are able to alleviate the effects of the disease [16,17].

As early as three decades ago, PDT began to be used successfully in the treatment of bladder cancer. The team of Prout et al. conducted a joint study to evaluate the effectiveness of this form of therapy in treating superficial transitional cell carcinoma of the bladder. They used a hematoporphyrin derivative as a photosensitizer and proved that PDT is useful in the treatment of this type of cancer [18].

The photosensitizer (PS), which is the most important element of PDT, plays a decisive role in PDT therapy. Zhao et al. summarized the recent advances made in developing PS for the treatment of cancer diseases. These advances include PSs designed to demonstrate increased tolerance to the tumor microenvironment, improved tumor specific selectivity and applicability in deep tissues [19].

## 2. Photodynamic Therapy (PDT) in General

PDT is light therapy of the appropriate wavelength used to remove cancerous tumors. It is often used in modern cancer therapy. It consists in administering a photosensitizing agent to the tumor area and then activating it by light energy of a specific wavelength. Light energy in the presence of oxygen produces a photodynamic reaction that is cytotoxic and vascularly toxic. PDT does not seriously damage normal tissue in the patient’s body. However, PDT selectivity is not absolute, and some damage can occur to the normal tissue [20].

The use of three non-toxic components is the basis for the operation of the PDT mechanism. Mutual interactions of the (PS), light of the appropriate wavelength and dissolved oxygen in the cells produce destructive effects within pathological tissues. There are two main mechanisms of photodynamic reaction and both are closely dependent on the oxygen molecules found inside the cells. The first stage is analogous for both mechanisms. The mechanisms of PDT are divided in two categories: type I and type II photoprocesses. Both Type I and Type II mechanisms have an absolute dependence on molecular oxygen Type II mostly involves energy transfer from the photosensitizer to ground-state ^3^O_2_ to yield singlet oxygen (^1^O_2_), and type I involves photoinduced electron transfer that leads to the formation of superoxide (O_2_^•−^) or hydroperoxyl radicals (HO_2_^•^). Both Type I and Type II photosensitized reactions result in biomolecule degradation and ultimately tissue damage/destruction [21].

**Figure 1:**
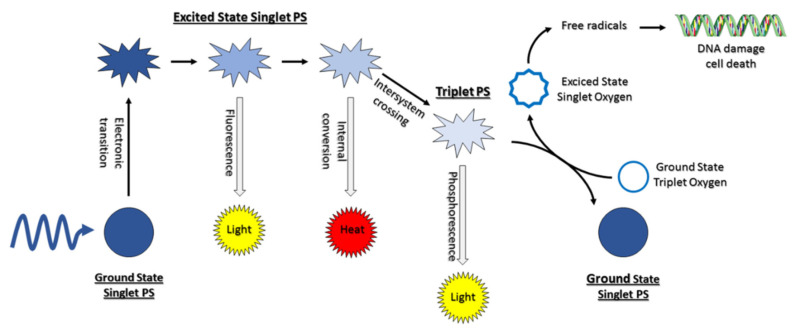
Molecular mechanism of PDT (adapted with permission [22]).

Namely, the photosensitizing agent, after entering the cell’s interior, is irradiated with light of appropriate wavelength, which corresponds to its absorption. The light-excited photosensitizer is transformed from the singlet ground energy state S° to excited singlet state S^1^ due to photon absorption. Then, some of the energy is radiated as fluorescence, and the PS may intersystem cross the excited state triplet (Figure 1) [22,23].

The photocytotoxic reactions take place only at the site of pathologically changed tissue, in the area where the photosensitizer is spread, allowing selective destruction of the site [24]. As has been shown in many studies, photosensitizers accumulate in higher concentrations in cancer cells than in healthy cells. This is related to the tendency of photosensitizers to associate with low-density lipoproteins (LDL). LDL lipoproteins are assigned the function of supplying tissues with the necessary cholesterol to build membranes of cells undergoing division. Rapidly dividing cancer cells are characterized by an increase in the concentration of LDL lipoproteins, which in turn are responsible for the transport of the photosensitizer to neoplastic tissues [25,26,27,28,29,30].

It has been shown that tissues composed of cells with increased cell division activity are characterized by high expression of LDL lipoprotein receptors on the cell surface. Thus, the affinity of photosensitizers to serum lipoproteins plays an important role in delivering these drugs to neoplastic tissue [31,32].

Numerous studies show that (PDT) influences the tumor vascular system and stimulates the body’s immune system. Neoplastic cells that survive by direct photocytotoxicity may still be destroyed by the indirect influence of PDT. Damage to vascular endothelial cells by reactive oxygen species activates the coagulation processes, platelet aggregation and closes the lumen of blood vessels, causing clots to form. Their formation leads to obstruction of blood vessels, which results in hypoxia of the neoplastic tissue and, consequently, leads to cell death [33,34]. The effect of PDT destroys the structure of the neoplastic tumor and stimulates the direct interaction of immune cells with cancer cells. It is now known that PDT leads to a systemic anti-tumor response.

## 3. Photodynamic Therapy in Bladder Cancer

PDT is a therapy that works very well in the treatment of bladder cancer. There are five excellent reviews that discuss PDT in bladder cancer photomedicine [35,36,37,38,39].

PDT has found wide application in medicine, in particular in neoplastic diseases, including bladder cancer. Numerous studies have shown that photosensitizing substances accumulate selectively at high concentrations in cancer cells and to a much lesser extent in healthy cells.

Photodynamic diagnostics (PDD) is an innovative unconventional technique. It is an integrative method because it takes advantage of the fact that PS is selectively absorbed intracellularly to a greater extent only by neoplastic cells in view of the physiology of neoplasms. The absorbed PS as a result of the exposure of laser light with a specific wavelength is activated, which causes the phenomenon of fluorescence. Cancer cells that have absorbed the photosensitizer emit light without damaging other organs. Thanks to this, it is possible to quickly and easily identify neoplastic changes. The PDD method enables easy visualization and identification of neoplastic cells, enabling early diagnosis and thus more effective treatment [40].

The bladder is accessible by endoscopy and the tumors are most often confined to the mucosa or submucosa. PDT is probably more useful for patients with recurrent tumors after conventional therapies, as well as for patients with diffuse non-invasive bladder cancers refractory to standard treatment prior to radical extirpative surgery, especially in patients at high surgical risk [41].

In bladder cancer photodiagnosis, diseased tissue containing PS is exposed to light of a certain wavelength. The photosensitizer accumulated in the cells causes the emission of radiation. The result is an image of red fluorescent tumor tissue surrounded by healthy tissue that is green in color. On the other hand, in photodynamic therapy, the task of the photosensitizer is not to visualize neoplastic cells, but to destroy them, caused by the products (reactive oxygen species) of the reaction taking place. After the administration of the photosensitizer and the exposure of tissues and cells to laser light of the appropriate wavelength (for a given photosensitizer), reactive oxygen species are produced, which indirectly destroy cancer cells, initiating apoptosis.

The PS can accumulate not only in cancer cells, but also in existing tissues in the inflammatory process. This can lead to an increase in the number of false positives. In the study by Aboumarzouk et al. [42], they noticed increased fluorescence less than 3 months after intravesical administration of BCG (Intravesical Bacillus Calmette-Guérin immunotherapy) and after removal of the bladder. BCG immunotherapy remains the most effective treatment and prophylaxis in superficial transitional cell carcinoma (TCC) and reduces tumor relapse, disease progression and mortality. Interferons, KLH (Keyhole-limpet Hemocyanin), Bropirimine and Photofrin-PDT are being studied in the treatment of TCC and the results obtained are encouraging [43]. It is worth discussing that TGF-β1 is a multifunctional cytokine and is synthesized by nearly all cells and affects many physiological processes; its particular cell-specific activity depends on the cell environment and is stored as a large latent complex in the extracellular matrix (ECM), waiting to be activated and to establish a mechanical checkpoint in the progression of tissue repair. TGF-β1 is a general suppressor of cell proliferation and has an immunosuppressive activity through different mechanisms [41]. Some authors have reported that growing tumor cells can also be the source of transforming growth factor, entailing a specific immunosuppression observed in patients with malignancies; others suggest that some members of TGF-β1 superfamily, such as activin A, might be associated with tumor aggressiveness [42]. Some authors describe a local TGF-β1 inhibition by PDT, preventing vascular smooth muscle injury [43]. Adamek and coworkers have observed a significant decrease of TGF-β1 serum level after the topical use of 10% delta-aminolevunic acid (an endogenous non-proteinogenic amino acid) and showed that the immune system response in patients undergoing PDT due to basal cell carcinoma (BCC) are promising [44].

Twenty weeks observation confirms good therapeutic effect in the inflammation of the glans in patient resistant to standard treatment involving antibiotics, steroids and cryosurgery. PDT particularly focusing on aminolevulinic acid (ALA) is called ALA—PDT. ALA—PDT offers a safe and cost-efficient treatment session. In addition to a favorable tolerability profile, most patients also achieved an improvement in skin texture. They observed significant improvement after first ALA—PDT, and after five procedures of ALA—PDT, the patient was found cured [45]. PDT in vivo is based on prevention of expansion and detection of recurrence of the cancer [46].

In addition, cryotherapy is a technique used to kill bladder cancer cells by freezing them. The advantages of PDT are connected with minimally invasive and localized character of the treatment and with not damage of collagenous tissue structures, therefore normal cells will repopulate these arrangements [47]. In addition, PDT due to toxic and local behavior of singlet oxygen may be an advanced treatment in antimicrobial therapy [37].

### 3.1. Using Different Synthetic Photosensitizers

One of the most important works with the use of a photosensitizer was the use of hematoporphyrins in the diagnosis of bladder cancer. Intravenous administration of a hematoporphyrin derivative to the patient resulted in fluorescence under UV light with a characteristic bright red color of the diseased tissue. Normal tissues are not fluorescent [48].

In the clinic with the use of PDT, several photosensitizers are used, both synthetic and of natural origin. The most common medical databases mention: ALA (5-aminolevulinic acid) [17,49,50,51,52,53] and its ester derivative—HAL (hexylaminolevulinate) [45,46,47]. (ALA) is known as a precursor in the biosynthesis of the naturally occurring porphyrin, haem. Haem is known to be a component of haemoglobin, myoglobin and other haemproteins. Protoporphyrin IX (PPIX) is known as a precursor to haem. Haem itself is not a photosensitiser, due to the coordination of a paramagnetic ion in the centre of the macrocycle, causing significant reduction in excited state lifetimes [49,50,51,52,53,54,55,56].

On the other hand, photosensitizers obtained from natural sources and having a positive effect are hypericin [57,58,59] and chlorophyllin [60,61]. Photosensitizers should absorb light in the red or far-red wavelengths in order to penetrate tissue deeper [49,50,51,52,53,54,62].

**Figure 2:**
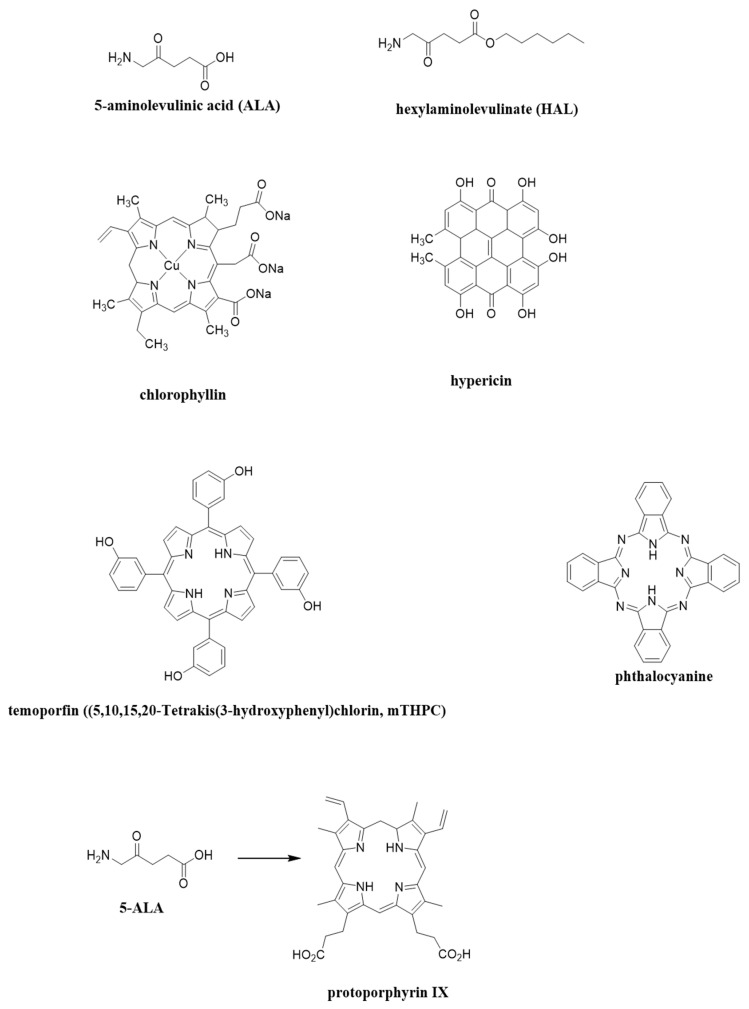
Photosensitizers structures discussed in this manuscript. Graphics prepared with ChemDraw^®^ Professional Perkin Elmer 2020.

Figure 2 shows the chemical formulas of the most commonly used photosensitizers in a doctor’s office. The selection was carried out by searching for naturally occurring compounds in plants or animals. ALA is a naturally occurring amino acid and HAL is naturally occurring heme precursor. Copper chlorophyllin is authorized as a natural green colorant. Hypericin is an anthraquinone derivative which is one of the principal active constituents of Hypericum (Saint John’s wort).

Looking for potential novel photosensitizers is a crucial first step in PDT studies because, to date, there are only a small number of approved PDT drugs, including Photofrin^®^, Foscan^®^ and Levulan^®^ which are used mainly for skin, gynecological, gastrointestinal, and head and neck cancers [63]. The advantages of PDT are minimal invasiveness, minimal toxicity short treatment time, and low cost. Therefore, new photosensitizers are constantly under Clinical trials. In March 2017, TLD1433 was administered to the first patient in a human clinical trial (ClinicalTrials.gov Identifier: NCT03053635). Tumor necrosis factor-related apoptosis-inducing ligand (TRAIL) is a promising candidate for anticancer therapy due to its ability to selectively induce apoptosis in cancer cells. However, not all tumor cells are sensitive to TRAIL. TRAIL resistant cancer cells can be sensitized to TRAIL induced apoptosis by anticancer agents. Effect of ALA-mediated PDT augments the cytotoxic effect of TRAIL on transitional cancer cells of bladder. The obtained results suggest that combined treatment of TRAIL and PDT may provide the basis for a new strategy to induce cytotoxicity in bladder cancer cells [64]. Improved diagnostic and therapeutic methods that aim to reduce rates of recurrence and progression of bladder cancer are needed. In current publications, one can find information on such methods as Raman spectroscopy, ultraviolet autofluorescence microscopy, confocal laser endoscopy, photoacoustic imaging, molecular imaging, multi-photon microscopy and many other new diagnostic techniques. These methods do not show significant adverse effects and are procedures well tolerated by patients as they use mostly physical phenomena that are neutral towards the human body [5]. In order to understand, transitional cell carcinoma (TCC) of the urinary bladder is nowadays one of the most common cancers in young men. Similarly, to other cancers TCC can be treated with curative intent when it is diagnosed very early. In recent years there has been an intensive development of treatment methods of urological diseases based on modern scientific discoveries, one of which is PDT [65]. The aim of the study was to evaluate the influence of (ALA) mediated photodynamic effect on secretory activity (MIF, MCP-1) of colon cancer cells in vitro both in normoxia and in hypoxia-like conditions [66]. ALA oral administration was used in transurethral resection of bladder tumor and was associated with hypotension during general anesthesia in patients who underwent these conditions [67]. Verteporfin (VP) is able to inhibit bladder cancer cell growth and invasion in a dosage dependent manner Purlin Chlorin is a tin etiopurpurin and has undergone Phase II clinical trials for cutaneous metastatic breast cancer and Kaposi’s sarcoma in patients with AIDS), also used to treat the non-malignant conditions psoriasis. Purlytin has been reported to localize in skin and produce a photoreaction 7–14 days post-administration. This PS is a meta-tetrahydroxyphenyl chlorin (mTHPC) and photoactivation at higher wavelengths (650–660 nm) provides high tumoricidal depth (10 mm) for PDT. Research was carried out on investigation of the photodynamic characteristics of mTHPC in solvent-based formulation (Foscan) and in liposomal (water soluble) formulation (Foslip) in an in vitro model system consisting of two biliary cancer cell lines (GBC, gall bladder cancer and BDC, bile duct cancer cells) [68,69]. Table 1 present current photosensitizers subjected to bladder cancer PDT [68,69,70,71].

#### 3.1.1. 5-Aminolevulinic Acid (ALA or Levlan)

Aminolevulinic acid (5-amino-4-oxopentanoic acid, commonly ALA) is an endogenous metabolite that is physiologically formed in the mitochondria. Absorption takes place at a light length of 635 nm. Eight ALA molecules after conjugation form the natural protoporphyrin IX (PpIX), which has photosensitizing properties. ALA—PDT has many advantages over traditional cancer treatments. It reduces long-term morbidity and offers the possibility of re-treatment [64,72].

PDT with ALA is a method of treatment in which the fluorescent substance protoporphyrin IX is excessively accumulated in the tumor cells. By irradiating with visible red or green light, the reactive oxygen produced in the process damages the cancer cells. 5-aminolevulinic acid PDT has less of an effect on surrounding healthy cells and tissues. Due to the lack of accumulation of protoporphyrin IX, it is minimally invasive.

The results of Bachor et al. show that ALA-induced photosensitization has a high potential for PDT of superficial bladder carcinoma [49]. A study by Kriegmair et al. in patients with bladder dysplasia and early-stage cancer using PDT has been shown extremely high sensitivity thanks to the use of (5-ALA). In patients with a planned bladder biopsy, ALA solution was administered directly into the bladder. A much higher sensitivity of this technique was observed (96.9%) than that of classic white light cystoscopy (72.7%) [73].

PDT with ALA is painless and does not require anesthesia. Local lesions are treated with low energy levels and are applied repeatedly, in contrast with radiation therapy. It is believed to be a new, minimally invasive treatment based on a concept different from previous treatments. In fact, PDT with ALA in the treatment of bladder cancer has been clinically demonstrated mainly for in situ refractory bladder cancer with favorable results [74]. PDT with ALA is photodynamic technologies based on the common biological characterization of cancers and is expected to be new therapeutic strategies for many types of cancer.

#### 3.1.2. Hexaminolevulinic Acid (HAL)

(PDT) with (ALA) is a highly selective treatment for malignant cells. PDT using ALA has the potential to be a therapeutic strategy for various types of cancer. PS absorption occurs at a wavelength of 635 nm light.

Studies assessed the safety and feasibility of (HAL)-based (PDT) as adjuvant therapy after transurethral resection of the bladder (TURB) in patients with moderate to high-risk urothelial bladder carcinoma (UCC). The procedure was performed in 17 patients who received the HAL solution intravenously. The bladder walls were irradiated. After time, the effectiveness of the PDT method was defined using cystoscopy, cytology and histology as the number of patients without a tumor. Initial efficacy results showed that of the treated patients, 9 were tumor free at 6 months, four were tumor-free at 9 months, and two were tumor-free at 21 months. PDT using HAL and an inconsistent white light system with a special irradiation catheter system has been found to be technically feasible and safe and may be an alternative treatment for medium to high-risk non-invasive bladder cancer [53].

A team of scientists from Romania presented a report based on the results of studies with the use of (HAL) in the treatment of non-invasive cancer of the bladder muscle. PDT with the use of HAL revealed 25.8% more tumors than classical cystoscopy. A significant reduction in disease relapse was also observed after the use of HAL [75].

Other studies showed that fluorescence cytoscopy (AC) using HAL improves the detectability of bladder cancer, especially in comparison with WLC (white light cystoscopy) [76,77].

Side effects from ALA or HAL therapy are rarely reported, e.g., painful urination, hematuria, bladder pain and bladder spasm. These symptoms are non-specific and are probably unrelated to drugs [49]. The results of only one study indicate the occurrence of anaphylactic shock after instillation of HAL in one patient [78].

ALA methyl ester and HAL, show an improved local bioavailability with respect to the parent compound, while conserving strong fluorescence induction, an effect attributed to the higher lipophilicity of the esters. ALA requires active transporters to reach the cytosol. ALA methyl ester is believed to follow active and passive transport mechanisms, while the higher lipophilicity (logP 1.8) and amphiphilic character of HAL enable it to diffuse through the cell membrane into the cytosol. This may explain why HAL induces cellular PPIX concentrations up to 50 fold above those reached with 5-ALA [79,80,81].

ALA or HAL induce porphyrin fluorescence in tumor spheroids cultured from several glioma cell lines, EMT 6 mammary carcinoma and bladder carcinoma cells, respectively [82,83,84].

Numerous photosensitizers have shown to be effective antimicrobial compounds in the treatment of gastric infectious diseases [85].

### 3.2. Herbal Photosensitizers

The market offers many compounds with photosensitizing effects. Much work on PDT has been published in recent years, but relatively little attention has been paid to natural extracts of medicinal plants and their compounds.

Herbal plants and their extracts are natural substances and are considered “green” compared to synthetic chemicals.

The role of plant extracts and natural compounds, i.e., hypericin and chlorophyllin, in the conduct of PDT used in bladder cancer is presented in this review.

#### 3.2.1. Hypericin

Hypericin is an anthraquinone derivative that is naturally extracted from *Hypericum perforatum*, otherwise known as the yellow flowering herb or commonly St. John’s wort. PDT with hypericin has been used to treat various types of cancer, including cancers of the skin [86], cervical [87,88], glioblastoma [89] and bladder [90]. PS absorption occurs at a wavelength between of 514–593 nm light.

Research reports that photoactivation of hypericin can generate superoxide anion radicals and singlet oxygen with good quantum efficiency. Tumors can be destroyed by reactive oxygen species (ROS) produced after PDT.

It has been found that activation of hypericin by light can inhibit protein kinase C (PKC) and a number of other growth factors, resulting in increased peroxidation of membrane lipids. This mechanism induces superoxide dismutase activity and is the reason for the reduction of cellular glutathione levels in the mitochondria [91].

The accumulation of cells in cancer cell lines has shown that hypericin localizes in the membranes of the nuclear envelope, the endoplasmic reticulum (ER), the Golgi complex, and also in the mitochondria. PDT therapy with hypericin clearly indicates that the main mechanism of cancer cell death is apoptosis, autophagy and necrosis [92,93].

Hypericin accumulates in the ER membrane leading to a rapid loss of Ca^2+^ stores, causing cell death by caspase-dependent or autophagy-dependent apoptosis. Another hypothesis in PDT with hypericin draws attention to the role of mitochondria, the damage of which may lead to the initiation of the intrinsic pathway of apoptosis. The mechanism involves the release of cytochrome c from the mitochondria, resulting in an increase in procaspase-9/procaspase-3 activation and poly ADP-ribose polymerase cleavage (PARP). The results presented may place hypericin as one of the most potent substances used as photosensitizers in PDT. Additionally, it is obtained from natural sources, which makes it cheap and readily available PS [94].

Other studies have been conducted on patients who underwent bladder cancer resection. These patients were administered a hypericin solution into their bladder. The results clearly indicate a higher sensitivity of the therapy [95]. A similar study with hypericin was also carried out by the team of Kubin et al. Patients with suspected or recurrent bladder cancer who underwent hypericin reported better results [96].

The team of Stavropoulos et al. used the polar methanol fraction of *Hypericum perforatum* L. extract as a photosensitizer for use in both PDT and photodynamic diagnostics (PDD). In the study, the efficacy of the extract as a phototoxic agent against bladder cancer was investigated using human bladder cancer cells T24 (high-grade metastatic cancer) and RT4 (low-grade primary papillary cancer). The photosensitizer at a concentration of 60 µg/mL after irradiation (with laser light with a wavelength of 630 nm) showed significant photocytotoxicity in both cell lines causing cell destruction from 80% to 86%. The lower concentrations (20 μg/mL) did not induce mortality in either cell line. The obtained results were compared with those in the same cell lines under the same conditions with the clinically approved Photofrin photosensitizer. Photofrin was used at the maximum clinically tolerated dose of 4 µg/mL, and was also excited with 630 nm laser light. In T24 cells, Photofrin showed slightly less photocytotoxicity (77%), in RT4 cells, Photofrin caused minimal cell death (9%) compared to the *Hypericum perforatum* L. extract. Cell death by the PDT action of methanol extract in these two bladder cell lines is mainly caused by apoptosis [97].

For even greater effectiveness, Bhuvaneswari et al. conducted research on the optimization of PDT protocols. The anti-tumor activity of hypericin PDT in combination with Erbitux (an angiogenesis inhibitor), which acts on the epidermal growth factor receptor (EGFR) in human bladder cancer cells, was investigated. The obtained results showed that the combination of Erbitux with hypericin-PDT strongly inhibited tumor growth in the bladder tumor model compared to the other groups. The tumor suppression was influenced by increased apoptosis as well as the identified ErbB4 dephosphorylation at the 1284 tyrosine site [98].

The reports described indicate significant photocytotoxicity, selective localization, easy and inexpensive extraction of the preparation. They emphasize that methanol extract can be an effective photosensitizer in PDT.

#### 3.2.2. Chlorophyllin

Chlorophyllin is a derivative of chlorophyll and is obtained from cyanobacteria and chloroplasts of algae and plants. Studies with its use have shown that chlorophyllin accumulates in lysosomes and mitochondria. Its localization site suggests that the main mechanism of chlorophyllin PDT in cancer cells is autophagy and apoptosis. Researchers also noted its favorable optical properties, ranging from 600 to 670 nm wavelength. Chlorophyllin is easily soluble in aqueous solutions, it is easy and cheap to obtain by extraction method compared to synthetic PS. It has low toxicity and is quickly removed from the body [71,72].

The authors of the study of chlorophyllin in the application of PDT have clearly discovered the anti-cancer effect. They found that chlorophyllin induces apoptotic death and autophagy in cells. The results suggest that chlorophyllin is a new, effective PS and, when combined with autophagy inhibitors, may be an attractive therapeutic strategy against non-invasive human bladder cancer in PDT [99].

Patients with non-invasive bladder cancer (NMIBC) often relapse after surgery due to incomplete resection and chemoresistance. The aim of the work of the team of Zhuo et al. was to evaluate the anti-tumor effect of PDT using chlorophyllin and bladder cancer human tumor models (T24 and 5637). The results clearly showed that chlorophyllin-PDT exhibits significant cytotoxicity in the cells used. Cell migration and thus invasion capacity was significantly reduced, cells showed the typical morphological changes in apoptosis. It was shown that the level of intracellular reactive oxygen species (ROS) was significantly increased, while the activity of superoxide dismutase (SOD) was significantly decreased in cells treated with chlorophyllin-PDT [100].

Chlorophyllin shows a clear photocytotoxic effect, is an effective PS and may be used therapeutically in the treatment of bladder cancer.

## 4. Autofluorescence Cystoscopy

The ability to detect and diagnose bladder cancer early and precisely is crucial for effective treatment. The aim of study was to assess the utility of optical biopsy performed with autofluorescence cystoscopy (AFC) using the Onco-LIFE system with numerical color values (NCVs) and by ALA/PDD. Histopathological examination of material obtained during TURBT and/or biopsy of the bladder was carried out. The presence of a muscle-invasive tumor increased the NCV by approximately 2.86 compared to healthy tissue. The rates of postoperative complications depend on the examining operator and are observed more often, as much as 65.7% during ALA/PDD. AFC with NCV using the Onco-LIFE system, as well as ALA/PDD are helpful tools for early diagnosis of bladder precancerous and cancer lesions and for performing targeted biopsies. A significant correlation was found between lesion NCV index and the grade of dysplasia or tumor malignancy. Tissue with inflammation, metaplasia, and healthy tissue demonstrated significantly lower mean NCV values. AFE with NCV have a significantly higher sensitivity than specificity. Low rates of postoperative complications are correlated to the experience of the endoscopist and with AFE/NCV in comparison of ALA/PDD.

**Figure 3:**
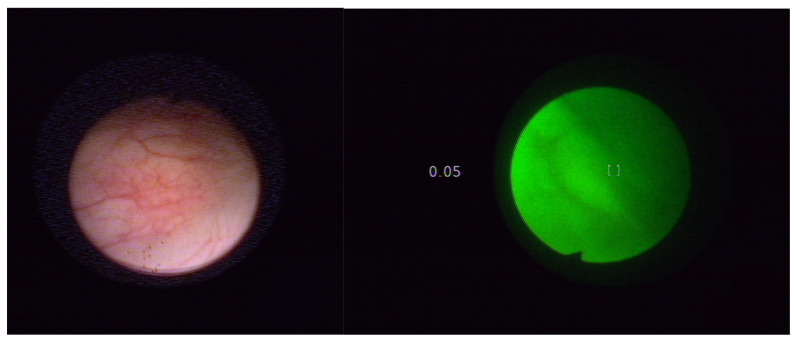
Normal bladder mucosa in white light and in autofluorescence with the Onco LIFE system. NCV-0.05 index (low-normal mucosa). Field of view at 3 cm of distance.

**Figure 4:**
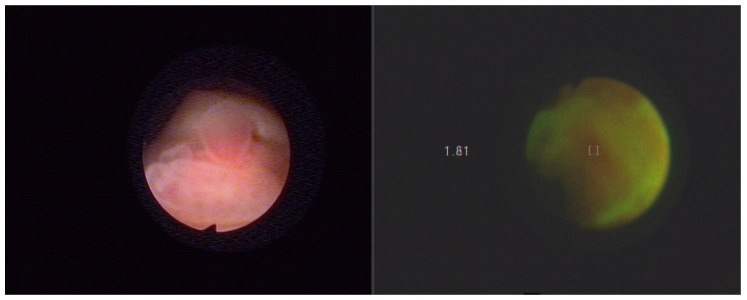
Bladder cancer in white light and in autofluorescence with the Onco LIFE NCV NCV system-numerical color value—1.81. Field of view at 3 cm of distance.

Endoscopic evaluation of the diagnostics included macroscopic lesions such as bladder tumors, squamous metaplasia, inflammatory lesions of scar tissue (after previous bladder procedures) and unchanged bladder tissue. Figure 3 presents Normal bladder mucosa in white light and in autofluorescence with the Onco LIFE system. Figure 4 presents bladder cancer in white light and in autofluorescence with the Onco LIFE NCV NCV system-numerical color value—1.81. The research was approved by the Bioethical Committee of the Medical University of Silesia on the basis of the Resolution KNW/022/KB1/46/15 of 25 May 2015. Images captured with the Xillix ONCOLIFE device are color maps composed of two channels R (red) and G (green). In the area of the red channel within healthy tissue a minimal grayscale signal is observed, while points are recorded within pathological tissues with the highest intensity in grayscale.

NCV + LIF/PDD diagnostics is a kind of “optical biopsy” of the tissue that is an alternative to invasive methods of cancer diagnosis. Both of these the methods use light in the blue wave range. Their advantages are: low invasiveness, good tolerance by patients, immediate results during the test and minimal side effects. As mentioned previously, the photosensitizer currently commonly used for PDD is ALA or HAL. Both agents ALA and HAL can be topically instilled into the bladder without causing any systemic or local toxicity. Differences in the application of both compounds are significant [73]. Therefore, in an application for optimal use, the following are given:

- ALA [(1.5 g or 180 mM) in 50 mL of sodium carbonate buffer solution], which must be instilled and retained in the bladder for 2–3 h prior to cystoscopy,
- HAL [(8 mM) in 50 mL of phosphate buffer solution], instilled and retained in the bladder for 1 h before [73].

## 5. Light Sources in Applications for PDD and PDT of Bladder Cancer

Lasers and other light sources convert the input energy into light. The laser has: an amplifying medium, a resonant cavity and an energy source. Currently, diode lasers are the most common light sources for clinical PDT therapy. Lasers can operate in pulsed and continuous modes. The most common types of lasers used in PDT are listed below. In a diode laser, light is generated as a result of the electron-hole interaction. A diode laser is a semiconductor device containing an amplifying medium and a resonant cavity. The source of energy is electricity. Diode lasers are small in size, light in weight, easily portable and inexpensive. In turn, dye lasers contain an organic dye molecule that emits light in a given spectral range. This makes it easier to match the absorption wavelength to a specific photosensitizer. The dye is in the form of a fluid with constant circulation. The PDT also uses fluorescent, incandescent, metal halide, xenon and sodium lamps. The spectrum of light generated by the lamp is wide, which is why optical filters are used in laboratories to adjust the wavelength to the appropriate absorption band of the photosensitizer [101].

In recent years, a great deal of research has been devoted to developing new measurement techniques for each of the quantities used in PDT (light dose, penetration and photosensitizer concentration). Various types of optical techniques are in use or under development to measure parameters in PDT. The light dose is measured mainly by optical fibers that can collect light at large solid angles. Tissue concentration of photosensitizer can be measured by positron emission tomography (PET) using labeled photosensitizers as detectable tracers. An alternative to PET is the fluorescence technique. This technique is based on measuring the emitted phosphorescence from singlet oxygen in the tissue as it returns to the ground state after excitation with the light used in PDT [102].

PDT offers the possibility of quantitative light dosimetry, e.g., in the treatment of superficial bladder cancer using focal or entire bladder wall irradiation. PDT is the local or systemic administration of a photosensitizing drug which, when irradiated with light and the presence of oxygen, causes tissue damage, such as tumor destruction. The clinical efficacy of PDT and PDD in the treatment of bladder cancer is dependent on the parameters of the irradiation source, such as bandwidth, maximum intensity and half width. The parameters of the irradiation source have to be used to optimize the main factors such as low intensity and low cost of the PDT or PDD. Therefore, a low light level is used prior to surgery to optimize the position of the light source. Parameters such as bladder volume, laser energy output, and desired light dose are important for adjusting the photon irradiation period to compensate for changes in laser light generation, energy losses during transmission, and changes in light intensity due to bladder action in terms of luminous flux produced. during photoirradiation.

Hypericin, is a hydroxylated phenanthroperylenquinone and is often used in bladder cancer research or clinic [103]. Hypericin in the bladder (30 mM) was irradiated using 595 nm laser light. This research demonstrated that light doses of 12–48 J/cm^2^ resulted in selective PDT-induced urothelial tumor damage without damaging detrusor musculature [104]. Hypericin (30 mM) in the human bladder tumors and normal bladder was detected using fluorescence microscopy. In situ quantification of Hypericin showed that there was much more Hypericin fluorescence in the tumor than in the normal bladder, with the tumor to normal bladder ratio mounting to 12:1 after 4 h of Hypericin applications [105]. Whole bladder wall PDT was performed using approximately 630 nm light emitted by an isotropic light source centered in the bladder cavity. The phenomenon of an increased fluence rate in this spherical geometry, due to light scattering, is denoted as the integrating sphere effect. The optical properties of cancer human bladder tissue, i.e., absorption coefficient, scattering coefficient, anisotropy factor and refractive index, were determined in vitro in the wavelength range of 450–880 nm [106]. The detection of the bladder by fluorescence endoscopy with hematoporphyrin derivative and argon ion laser was performed using a video monitoring system coupling with an image intensifier; however, it was difficult because of the reflecting light of the excitation light. Whole bladder wall photoradiation therapy was carried out in 3 patients with multicentric CIS of the bladder, using argon dye laser light (630 ± 1.6 nm) of 5 to 25 J/cm^2^ [107]. In the research carried out by Marynissen and coworkers an isotropic light detector (0.8 mm. diameter probe on 200 microns. fiber) connected to an amplifier displaying light dose rate (in mW/cm^2^) and integrated light dose (in J/cm^2^) was used for whole bladder wall PDT with in vivo monitoring and control of light dose rate and dose. As per this experiment for the red light (wavelength 630 nm) a dose rate uniformity of ±20% was achieved in vivo in dog bladder. With green light (wavelength 514.5 nm) uniform irradiation was difficult, most likely due to a much smaller contribution of scattered light [108]. Hematoporphyrin derivative and the red light (wavelength 630 nm) and an argon-dye laser were used to perform PDT on 46 patients with superficial bladder tumors. Hematoporphyrin derivative (2–4 mg/kg) was i.v. injected 48 to 72 h before PDT. The light power was 200 mW/cm^2^ for 5–10 min or more and the total light energy should be 100 J/cm^2^ or more in tumors up to 2 cm in size [109]. Bladder tissue is relatively more translucent than other human tissues and there is therefore great potential for PDT in the treatment of bladder cancer [110].

## 6. Summary

In conclusion, in this review, we provided up-to-date information on the use of PDT as a therapeutic approach to bladder cancer. Much information highlights the importance of PDT as an alternative therapeutic approach that can induce several mechanisms of cell death due to the photosensitizer used. In the context of PDT, the major routes to cell death are apoptosis and shutdown of the tumor vasculature. Autophagy is usually a cytoprotective mechanism. Necrosis is generally avoided since the high light doses can cause photodamage to normal cell types.

PDT is an interesting technique, thanks to its use we can reduce the mortality of cancer patients, including those with bladder cancer. One feature that is important for alternative cancer therapy is the broadening of the spectrum of cell death mechanisms that are combined to bypass various malignant cell resistance mechanisms.

More research is needed in this field, including optimization of PS synthesis to increase efficacy against specific tumor cell damage, light sources and amount of irradiation.

PDT is in most cases a suitable therapeutic alternative, with several advantages over traditional clinical approaches to treating cancer. It is clear that PDT deserves more investment in clinical research, especially for plant-based photosensitizers. Natural PS isolated from plants and other biological sources can be considered a green approach to PDT in cancer therapy. Low systemic cytotoxicity to normal cells and selective action against malignant cells are some of the main advantages of natural PS in PDT. PDT, in combination with other natural compounds, fights malignant cells using three main components, i.e., PS (derived from plants), light and oxygen. All these components promote photochemical reactions that lead to the production of reactive oxygen species in malignant cells and, consequently, to cell death by inducing apoptosis. Research results confirm that natural compounds can be considered promising candidates for PDT. Moreover, because natural compounds are usually ubiquitous, they may be more readily available compared to synthetic chemotherapeutic agents. In addition, the use of natural PSs in PDT usually causes fewer or negligible side effects, such as erythema or swelling than other routinely used medications.

Currently, PDT is widely used in the treatment of skin cancer, but numerous studies show the advantages of related therapeutic strategies that can help eliminate various types of cancer, including bladder cancer.

PDT can become the standard of first-line therapy, alone or in combination with other treatments, for many different types of cancer.

PDD and PDT are of increasing interest in urology, especially in bladder cancer. The growing popularity of PDD and PDT applications in urology creates a new possibility in diagnostics and therapy. Photodynamic diagnostics offers hope for the improvement of diagnostics in patients with bladder cancer. Moreover, the combination of PDT with cystoscopy enables the non-invasive reduction of the number of cancer cells. Current experience with PDT using approved PS will allow that the new way of PDT application will lead through new form of PS designed for bladder cancer.

## Figures and Tables

**Table 1:** PS subjected to bladder cancer PDT.

Drug or Substances in	PS	Reference
Metvix^®^	methyl ALA ester	[70]
Benvix^®^	benzyl ALA ester	[70]
Hexvix^®^	hexyl ALA ester	[70]
Visudyne^®^	benzoporphyrin Verteporfin derivative monoacid ring A	[68,69,70]
Radachlorin^®^	3 chlorophyll a derivatives in an aqueous solution	[71]

## Data Availability

Not applicable.
